# Newly diagnosed HIV and use of HIV-PrEP among non-western born MSM attending STI clinics in the Netherlands: a large retrospective cohort study

**DOI:** 10.3389/fpubh.2023.1196958

**Published:** 2023-06-19

**Authors:** Ymke J. Evers, Cornelia J. D. Goense, Christian J. P. A. Hoebe, Nicole H. T. M. Dukers-Muijrers

**Affiliations:** ^1^Department of Sexual Health, Infectious Diseases and Environmental Health, Living Lab Public Health, South Limburg Public Health Service, Heerlen, Limburg, Netherlands; ^2^Department of Social Medicine, Care and Public Health Research Institute (CAPHRI), Maastricht University, Maastricht, Netherlands; ^3^Department of Medical Microbiology, Infectious Diseases and Infection Prevention, Care and Public Health Research Institute (CAPHRI), Maastricht University Medical Center+ (MUMC+), Maastricht, Limburg, Netherlands; ^4^Department of Health Promotion, Care and Public Health Research Institute (CAPHRI), Maastricht University, Maastricht, Netherlands

**Keywords:** HIV, PrEP, men who have sex with men, migrant, non-western, inequity

## Abstract

**Introduction:**

The World Health Organization recommends HIV-PrEP for all people at risk for HIV infection, which includes men who have sex with men (MSM). Substantial part of new HIV diagnoses in the Netherlands are in non-western born MSM. This study evaluated new HIV diagnoses and reported PrEP use among non-western born MSM and compared it to western-born MSM. To inform public health efforts in the context of equitable PrEP access, we further assessed sociodemographic factors related to higher HIV risk and lower PrEP use among non-western born MSM.

**Methods:**

Surveillance data of consultations among MSM in all Dutch STI-clinics (2016–2021) were analyzed. STI-clinics provide PrEP via the national pilot-program since August 2019. In non-western born MSM (born in Eastern-Europe/Latin-America/Asia/Africa/Dutch-Antilles/Suriname), sociodemographic factors were evaluated for association with HIV (by multivariable generalized estimating equations) and reported PrEP use in the past 3 months (by multivariable logistic regression; restricted to an at-risk for HIV person-level data-subset from August 2019).

**Results:**

New HIV infections were diagnosed among 1.1% (493/44,394) of non-western born MSM-consultations (vs. 0.4% among western-born MSM, 742/210,450). Low education (aOR: 2.2, 95%CI: 1.7–2.7, vs. high education) and age under 25 years (aOR: 1.4, 95%CI: 1.1–1.8, vs. age above 35 years) were associated with new HIV diagnoses. PrEP use in the past 3 months was 40.7% in non-western born MSM (1,711/4,207; 34.9% among western-born MSM, 6,089/17,458). PrEP use was lower among non-western born MSM aged under 25 years (aOR: 0.3, 95%CI: 0.2–0.4), living in less urban areas (aOR: 0.7, 95%CI: 0.6–0.8), and having low education level (aOR: 0.6, 95%CI: 0.5–0.7).

**Conclusion:**

Our study confirmed that non-western born MSM are an important key population in HIV prevention. Access to HIV prevention, including HIV-PrEP, should be further optimized to all non-western born MSM at risk for HIV, and specifically to those who are younger, live in less urban areas, and have a low education level.

## 1. Introduction

HIV pre-exposure prophylaxis (PrEP) is a highly efficacious and cost-effective HIV prevention strategy, that reduces HIV incidence. Therefore, the World Health Organization has recommended PrEP for all people at substantial risk for HIV infection (1). Many high-income countries have taken up PrEP into their public health response to HIV by reimbursing PrEP (covering costs by government and/or insurances) for people at high risk of HIV acquisition. In these countries, men who have sex with men (MSM) have been identified as a priority population for PrEP. In the Netherlands, 63% of new HIV-infections have been diagnosed among MSM in 2020 (2). Persons from HIV-endemic regions, such as people from sub-Saharan Africa, South America and Southeast Asia, who are living in high-income countries, are also regarded a key population for HIV-prevention and care. Almost half (42%) of people diagnosed with HIV at 15 years of age or older were born outside of the Netherlands. Although a substantial part are pre-arrival HIV diagnoses, 61% of those diagnosed in 2018 or later reported having acquired their HIV infection in the Netherlands (2). Of MSM being diagnosed with HIV, 71% originated from the Netherlands, 10% from other European countries, 7% from South America, and 4% from the Caribbean. In recent years (i.e., for diagnoses in or after 2018), the proportion of MSM of Dutch origin was 63%, while slight increases were observed in the proportion of MSM from central Europe, South America and the Caribbean (2). This underpins the importance of PrEP accessibility for non-western born MSM at high risk of HIV acquisition. Internationally, there are to date (by our knowledge) no published data on uptake of PrEP by non-western born MSM in a country where PrEP is reimbursed.

In accessing PrEP, key are organizational, and social and personal factors. Several barriers have been identified for PrEP uptake among migrants in high-income countries, including HIV-related stigma, barriers to openly discuss sexual health, and non-equitable access (3). In the Netherlands, the PrEP-pilot program is primarily organized by STI clinic of the Public Health Services. Currently, the program allows only a restricted number of patients, resulting in a waiting list.

Here, we evaluated new HIV diagnoses and reported PrEP use in non-western born MSM who attended a Dutch STI clinic, and compared it with western-born MSM. To inform public health efforts in the context of equitable PrEP access, we further assessed sociodemographic factors related to higher HIV risk and lower PrEP use among non-western born MSM.

## 2. Methods

### 2.1. Study design

In this retrospective cohort study, coded surveillance consultations of MSM were included from all outpatient STI clinics in the Netherlands (25 Public Health Services with 38 STI clinic locations) which were submitted between 1 January 2016 and 31 December 31 2021 via an electronic patient registry using a consultation code to the National Institute of Public Health and the Environment. From 2016, individual-level data (with a patient identifying number) was available. Reporting to this national institute is standardized and mandatory for all STI clinics. For the current study, consultation-level and individual-level data in MSM was extracted on sociodemographic characteristics, sexual behavior in the past 6 months, STI and HIV diagnoses, and PrEP use in the past 3 months.

### 2.2. Study context

STI clinics provide free of charge, anonymous and confidential sexual healthcare to STI high-risk groups, including MSM, young people under the age of 25, and individuals from STI-endemic countries. All MSM are routinely tested for urogenital, anorectal and oropharyngeal *Chlamydia trachomatis* (CT), *Neisseria gonorrhoeae* (NG), HIV, hepatitis B and syphilis as part of routine sexual healthcare. MSM are generally advised to test every half year for STIs and HIV; and MSM using PrEP are advised to test every 3 months for STIs and HIV. Urine, self-collected anorectal swabs and nurse-collected oropharyngeal swabs were tested for CT and NG. Venepuncture nurse-collected blood samples were tested for HIV, hepatitis B and syphilis by screening and confirmatory tests. Specimens were tested in regional laboratories using different commercially available diagnostic assays. All tests were performed according to the manufacturers’ protocol. Further, STI clinics have been providing PrEP care via the national PrEP pilot program since August 2019 to MSM with a high risk of acquiring HIV (4). This high risk is translated as having condomless anal sex with people of whom the HIV-status is unknown or with people who are not adequately treated for HIV. PrEP care (STI tests and medical check-ups) is reimbursed from the program, but people have to pay a personal contribution of €7.50 for 30 PrEP pills. The program has a restricted number of available places and has a waiting list. It was estimated that more than 3,000 people eligible for PrEP were on this waiting list based on consecutive enrollment, with a varying waiting time of a few months to more than a year depending on the Public Health Service region (February 2023, the Netherlands). In the Netherlands, people are also offered PrEP care at the several general practitioners (GPs; not all GPs offer PrEP care). At the GP, PrEP care is more expensive, as people have to pay between €30–€50 per month for PrEP pills and have to pay themselves for STI tests and medical check-ups.

### 2.3. Variables and definitions

MSM were defined as men who reported having sex with men in the past 6 months. A new HIV diagnosis was defined as a positive HIV test in consultations of individuals with no previous (registered or reported) positive HIV test. Recent PrEP use was defined as reporting PrEP use in the past 3 months at the time of the consultation. Non-western born migrant was defined as a person born in Eastern Europe, Latin America, Asia, Africa, Netherlands Antilles or Suriname. Western-born was defined as MSM born themselves and of whom parents were born in Europe, Oceania or North America. Age was grouped into <25, 25–35, >35 (based on tertile distribution). Urbanicity was based on postal codes and categorized into high urban (≥1,500 addresses/m2) and low urban (<15.00 addresses/ m2) according to Statistics Netherlands.^1^ For consultations with missing postal codes, urbanicity was determined based on level of urbanicity of the Public Health Service region. Educational level was categorized into low: no education, elementary, pre-vocational secondary, senior general secondary, pre-university, and secondary vocational; and high: higher professional, university.

### 2.4. Statistical analyses

Proportion of new HIV diagnosis was compared between non-western born and western-born MSM consultations using descriptive statistics and chi-square tests. The proportion of new HIV diagnoses among western-born MSM was used as a reference proportion. Descriptive statistics and chi-square tests were used to assess differences in proportions of new HIV diagnoses by different continents of birth among non-western born MSM. For the outcome new HIV diagnoses, univariable and multivariable generalized estimating equations (GEE) analyses were used to assess independent sociodemographic factors related to new HIV diagnoses in all consultations in non-western born MSM. Assessed factors were age groups, urbanicity and educational level, and continent of birth. In multivariable analyses, all factors were entered and associations were adjusted for time calendar period based on the start of the PrEP pilot (before and after August 2019). GEE analyses were used to control for repeated measurements of patients that visited the STI clinic multiple times during the study period, using a non-identifiable patient identifier.

For the outcome PrEP use, analyses were performed on a subset of the data to reflect only PrEP-eligible persons. Therefore, we included the most recent consultations of MSM, who reported at least three sex partners in the past 6 months without consistent condom use during anogenital sex, in the period between 1 August 2019 and 31 December 2021. Proportion of PrEP use was compared between non-western born and western-born MSM using descriptive statistics and chi-square tests. The proportion of PrEP use among western-born MSM was used as a reference proportion. Descriptive statistics and chi-square tests were used to assess differences in proportions of PrEP use by different continents of birth among non-western born MSM. Univariable and multivariable logistic regression analyses were used to assess independent sociodemographic factors related to recent PrEP use among non-western born MSM. Heterogeneity of associations with continent of birth were tested for various sociodemographic groups (age groups, urbanicity) by including interactions terms into the models. In a sensitivity analysis, associations between new HIV and sociodemographic factors were assessed by multivariable logistic regression analyses on the person-level high-risk data subset from 2019. *p*-values < 0.05 were considered statistically significant. All analyses were performed using SPSS V27 IBM Corp., Armonk, NY, United States.

### 2.5. Medical ethics

The Medical Ethics Committee of Maastricht University waived the requirement for ethical approval and written informed consent because the data were coded, originated from standard care, and were analyzed anonymously (METC 2017-0251).

## 3. Results

### 3.1. Study population

**Figure 1:**
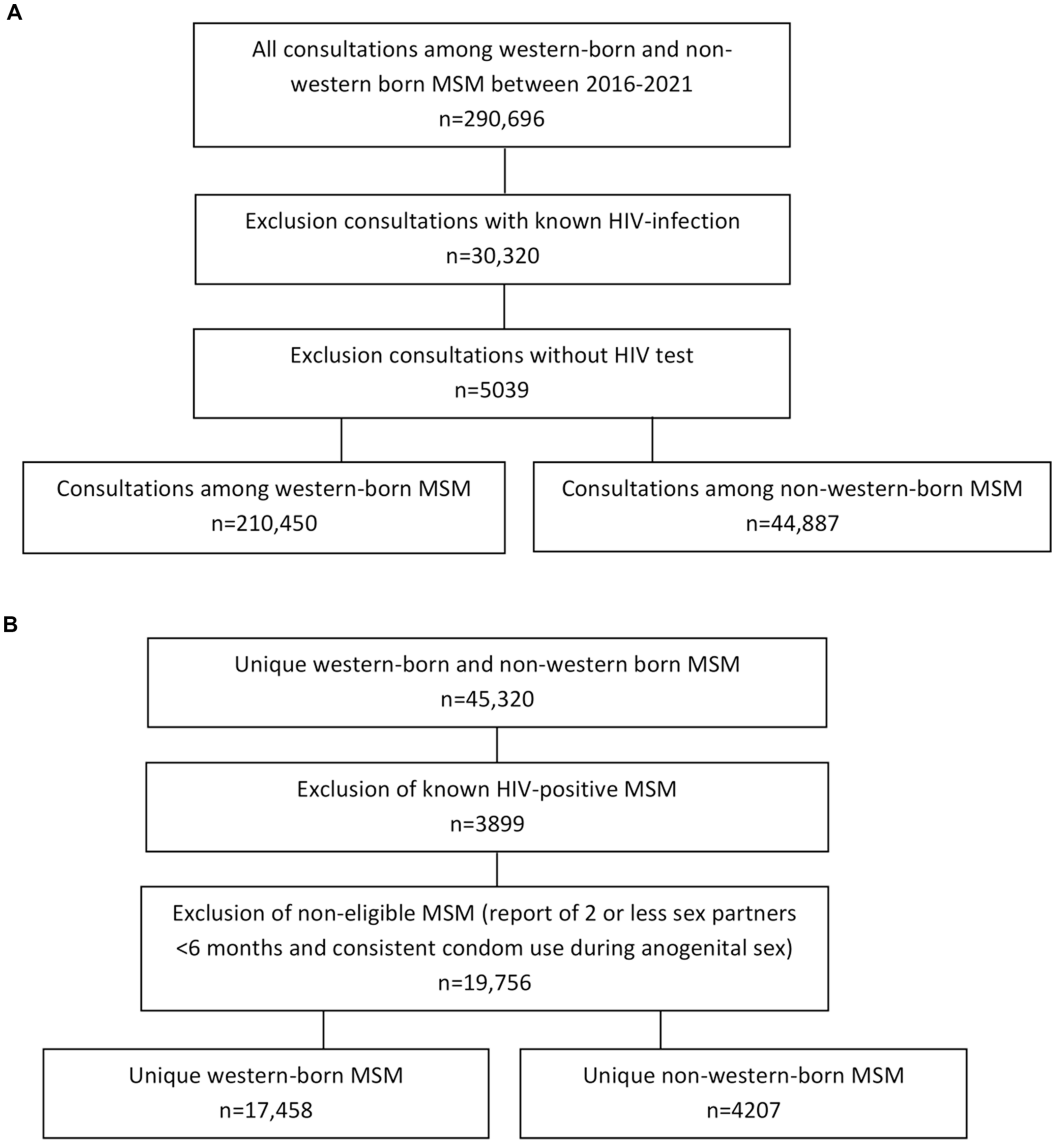
Flowchart of the study population. **(A)** Dataset used to assess proportion of new HIV diagnoses among western-born and non-western born MSM consultations in the period between 1 January 2016 and 31 December 2021. **(B)** Dataset used to assess proportion of PrEP use among western-born and non-western born unique MSM (most recent consultations) in the period between 1 August 2019 and 31 December 2021.

**Figure 2:**
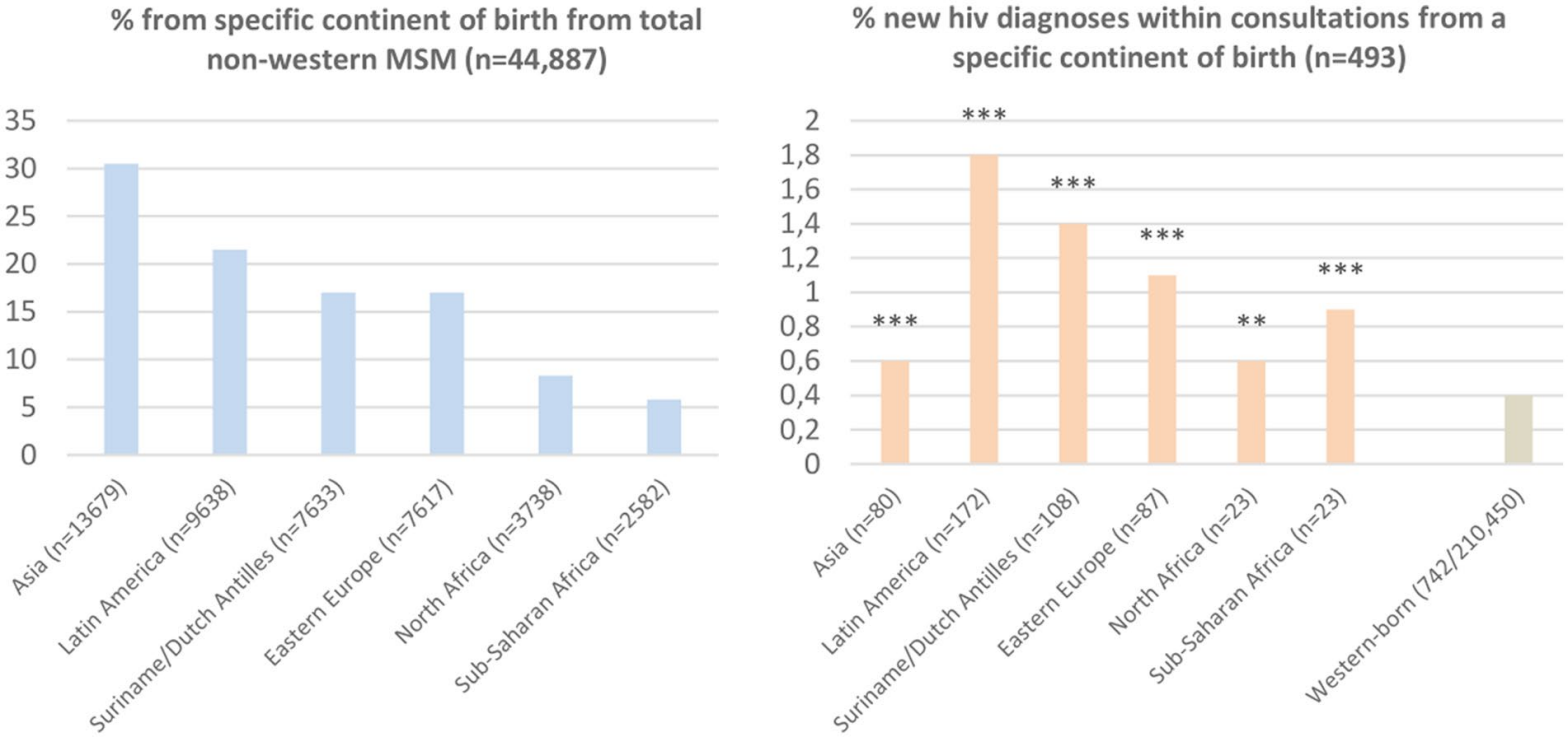
Proportion MSM from different continents of birth and new HIV diagnoses by continents of birth. Reference group western-born includes MSM born in the Netherlands (90.2%; 189,927), North/Western/Middle Europe (4.7%; 9,967), South Europe (3.5%; 7,404), North America (1.2%; 2,547), Oceania (0.3; 605). *** indicates value of *p* <0.001; ** value of *p* <0.01, compared to reference group Western-born by Chi-square tests.

After exclusion of consultations in which a known HIV-infection was reported and exclusion of consultations without a HIV test, the study population included 44,887 consultations among non-western born MSM and 210,450 consultations among western-born MSM (Figure 1). Median age among non-western born MSM was 32 years (IQR: 27–39) and 35 years (27–48) in western-born MSM. A high educational level was reported by 57.3% (25,737/44,887) non-western born MSM (26.9% low education level; 15.7% unknown educational level) and by 62.5% (131,620/210,450) western-born MSM (30.8% low educational level; 6.7% unknown educational level). Non-western born MSM were more often living in high urban areas (91.1%, 40,800/44,887) than western-born MSM (75.5%, 158,868/210,450). Among non-western born MSM, the most reported continents of birth were Asia, Latin America, Suriname/Dutch Antilles, and Eastern Europe (Figure 2).

### 3.2. New HIV diagnoses in non-western born MSM by continent of birth

New HIV was diagnosed among 1.1% (493/44,887) of non-western born MSM consultations (vs. 0.4%; 742/210,450 in western-born MSM). HIV positivity rate ranged from 0.6% in MSM born in Asia to 1.8% in MSM born in Latin America (Figure 2).

### 3.3. Factors associated with new HIV diagnoses among non-western born MSM

Proportion of new HIV diagnoses was higher among non-western born MSM aged under 25 years, having a low education level, MSM from Latin America, Suriname/Dutch Antilles, Eastern Europe (compared to Asia), and in consultations with missing postal codes (unknown urbanicity). All these factors were independently associated with new HIV diagnoses (Table 1). The association between age groups and new HIV diagnoses different significantly for continents of birth (significant interaction-term age and continent of birth, *p* = 0.012). Being aged < 25 years (aOR: 2.3, 95%CI: 1.4–3.8), and being aged between 25 and 35 years (aOR: 1.7, 95%CI: 1.2–3.5) were only significantly associated with new HIV diagnoses in MSM born in Latin America.

**Table 1:** New HIV diagnoses among non-western born MSM consultations, compared between sociodemographic groups by descriptive statistics and univariable and multivariable generalized estimating equations (GEE).

	New HIV diagnosis *N* = 493/44,887*%*	Univariable GEE analysis	Multivariable GEE analysis^#^
	*(n/N)*	*OR (95% CI)*	*aOR (95% CI)*
**Age group**			
<25	1.4 (91/6,401)	1.4 (1.1–1.9)^**^	1.4 (1.1–1.8)^*^
25–35	1.1 (248/22,596)	1.1 (0.9–1.4)	1.2 (0.9–1.5)
>35	1.0 (154/15,890)	1 (ref)	1 (ref)
**Urbanicity^**			
Low	1.2 (47/3,988)	1.1 (0.8–1.5)	1.0 (0.7–1.4)
High	1.1 (446/40,899)	1 (ref)	1 (ref)
**Educational level**			
Low	1.5 (182/12,089)	2.4 (1.9–2.9)***	2.2 (1.7–2.7)^***^
High	0.6 (166/25,737)	1 (ref)	1 (ref)
Unknown^&^	2.1 (145/7,061)	3.1 (2.5–3.9)**	3.1 (2.5–4.0)^**^
**Continent of birth**			
Latin America	1.8 (172/9,638)	3.1 (2.3–4.1)^***^	2.9 (2.2–3.8)^***^
Suriname/Dutch Antilles	1.4 (108/7,633)	2.5 (1.8–3.3)^***^	2.4 (1.8–3.2)^***^
Eastern Europe	1.1 (87/7,617)	1.9 (1.4–2.7)^***^	1.9 (1.4–2.6)^***^
Sub-Saharan Africa	0.9 (23/2,582)	1.5 (0.9–2.4)	1.3 (0.8–2.1)
North Africa	0.6 (23/3,738)	1.1 (0.7–1.7)	0.9 (0.6–1.6)
Asia	0.6 (80/13,679)	1 (ref)	1 (ref)

***indicates value of *p* < 0.001; **value of *p* < 0.01; *value of *p* < 0.05. ^Postal code was missing in 7% of consultations (3,229/44,887). For these consultations, urbanicity was based on level of urbanicity of the Public Health Service region. ^&^More than 5% missing values: a separate category unknown was included in the analyses. ^#^All variables were entered in multivariable model; model was adjusted for time period (before August 2019/From August 2019).

### 3.4. Study population in data subset to assess PrEP use

Exclusion of known HIV positive MSM and MSM reporting 2 or less sex partners in the past 6 months and consistent condom use during anogenital sex since they were ineligible to receive PrEP, resulted in 4,207 non-western born MSM and 17,458 western-born MSM who visited a Dutch STI clinic between August 2019 till 2021 (Figure 1). Median age was 32 years (IQR: 27–38) among non-western born MSM and 34 years (27–48) among western-born MSM. A high educational level was reported by 58.9% (2,476/4,207) non-western born MSM (25.8% low educational level; 15.4% unknown educational level) and by 64.4% (11,244/17,458) western-born MSM (30.2% low educational level; 5.4% unknown educational level). Non-western born MSM were more often living in high urban areas (90.8%; 3,819/4,207) compared to western-born MSM (75.8%; 13,226/17,458).

### 3.5. Current PrEP use among non-western born MSM by continent of birth

**Figure 3:**
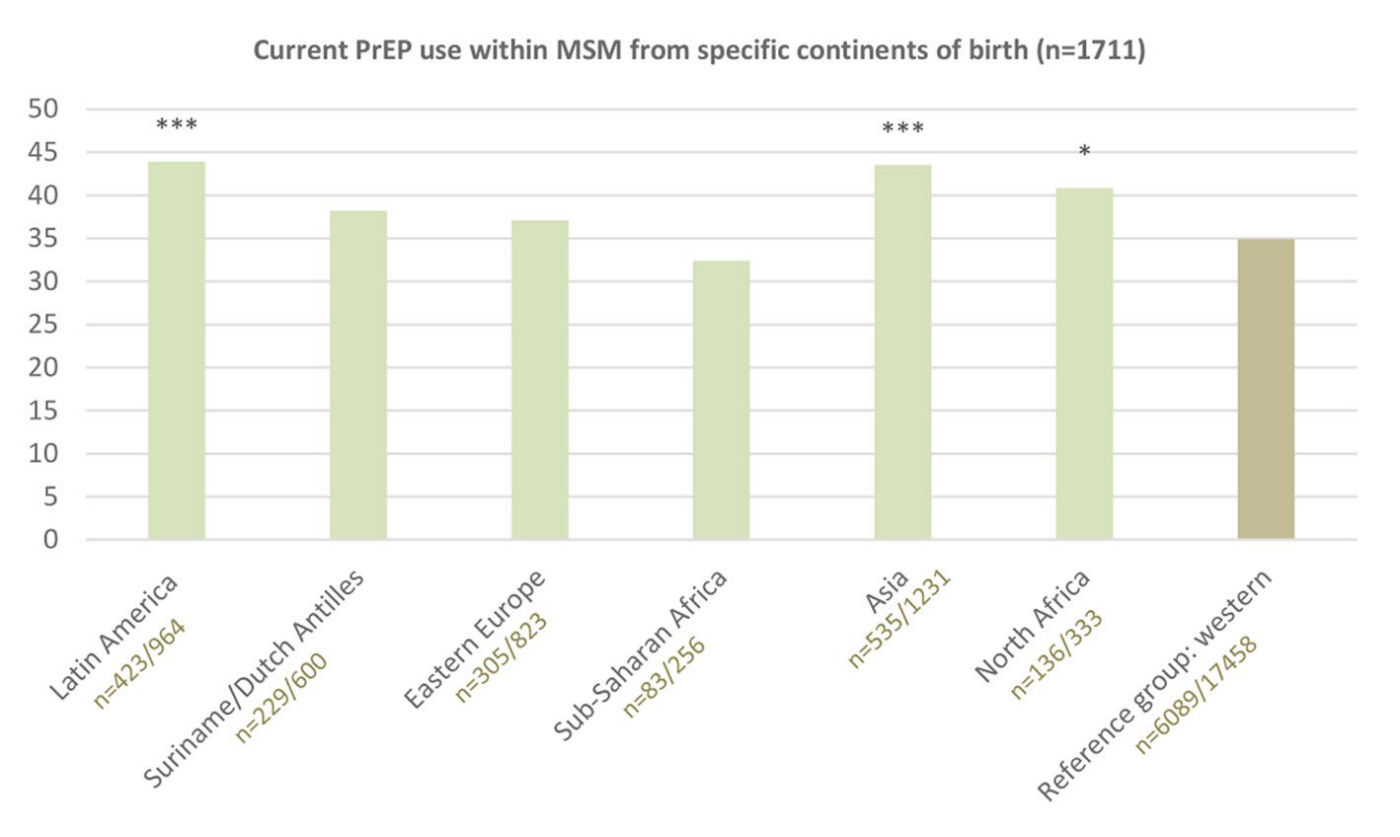
Reported PrEP use in the past 3 months by continents of birth. Reference group western includes MSM from the Netherlands (88.6%; 15,472), North/Western/Middle Europe (5.5%; 967), South Europe (4.0%; 698), North America (1.5%; 264), Oceania (0.3%; 57). *** indicates value of *p* <0.001; * value of *p* <0.05, compared to reference group Western-born by Chi-square tests.

PrEP use in the past 3 months was reported by 40.7% (1,711/4,207) of non-western born MSM (vs. 34.9% (6,089/17,458) of MSM born in Europe) during the most recent consultation (Figure 3). PrEP use ranged from 32.4% in MSM born in Sub-Saharan Africa to 43.8% in MSM born in Latin America. The median number of sex partners in the past 6 months reported by non-western born MSM reporting PrEP use was 8 (IQR: 5–15).

### 3.6. Factors associated with current PrEP use among non-western born MSM

Current PrEP use was lower in non-western born MSM aged 35 years or younger, who lived in low urban areas, had a low educational level, or were born in Suriname/Dutch Antilles, Eastern Europe or Sub-Saharan Africa. All these factors were independently associated with PrEP use (Table 2).

**Table 2:** Reported PrEP use in the past 3 months among unique non-western born MSM (most recent consultations in the period between 1 August 2019 and 31 December 2021), compared between sociodemographic groups by descriptive statistics and univariable and multivariable logistic regression analyses.

	PrEP use *N* = 1,711/4,207	Univariable logistic regression analysis	Multivariable logistic regression analysis
	*% (n/N)*	*OR (95%CI)*	*aOR (95%CI)*
**Age group**			
<25	21.6 (127/589)	0.3 (0.2–0.4)^***^	0.3 (0.2–0.4)^***^
25–35	40.9 (887/2,168)	0.8 (0.7–0.9)^***^	0.7 (0.6–0.8)^***^
>35	48.1 (697/1,450)	1 (ref)	1 (ref)
**Urbanicity^**			
Low	30.4 (118/388)	0.6 (0.5–0.8)^***^	0.7 (0.5–0.9)^**^
High	41.7 (1,593/3,819)	1 (ref)	1 (ref)
**Educational level**			
Low	32.0 (347/1,085)	0.6 (0.5–0.7)^***^	0.6 (0.5–0.7)^***^
High	45.5 (1,126/2,476)	1 (ref)	1 (ref)
Unknown^^&^^	36.8 (238/646)	0.7 (0.6–0.8)^***^	0.7 (0.6–0.8)^**^
**Continent of birth**			
Latin America	43.9 (423/964)	1.2 (1.0–1.3)^*^	0.9 (0.8–1.2)
Suriname/Dutch Antilles	38.2 (229/600)	0.9 (0.8–1.0)	0.8 (0.6–0.9)^*^
Eastern Europe	37.1 (305/823)	0.8 (0.7–0.9)^***^	0.8 (0.7–0.9)^*^
Sub-Saharan Africa	32.4 (83/256)	0.7 (0.5–0.8)^***^	0.7 (0.5–0.9)^**^
North Africa	40.8 (136/333)	1.0 (0.8–1.2)	0.8 (0.7–1.1)
Asia	43.5 (535/1,231)	1 (ref)	1 (ref)

***indicates value of *p* < 0.001; **value of *p* < 0.01; *value of *p* < 0.05. ^^^Postal code was missing in 8% of consultations (336/4,207). For these consultations, urbanicity was based on level of urbanicity of the Public Health Service region. ^&^More than 5% missing values: a separate category unknown was included in analyses.

## 4. Discussion

This study includes one of the first large scale evaluations of PrEP use among non-western born MSM who live in a high-income country with an available PrEP-program. We assessed that non-western born MSM generally have a higher proportion of new HIV diagnoses (1.1% vs. 0.4% in western-born MSM), and also use PrEP (41% vs. 35%) more often than MSM born in western countries (among those who are PrEP-eligible). However, several subgroups within non-western born MSM who have a higher or equal proportion of new HIV are less likely to use PrEP. These groups include younger people, people living in less urban areas and people with a low educational level. This indicates that efforts are needed to target both organizational and personal/social factors to promote access to PrEP care in these subgroups.

People born in all assessed non-western continents had a higher proportion of new HIV diagnoses than people born in western countries; highest proportions were noted in MSM born in Latin America, Suriname/Dutch Antilles and Eastern Europe. This is consistent with previous reports on HIV incidence among migrants living in Europe from non-western countries (2, 4). Our study suggests that PrEP use in PrEP-eligible MSM is higher in those born in Latin America, and not significantly different in those born in Eastern Europe and Suriname/Dutch Antilles compared to use in PrEP-eligible western-born MSM. For optimal individual (prevention of HIV) and population health outcomes (reduced transmission) equitable access to PrEP and uptake of PrEP for people with high HIV risk should be ensured. A previous Dutch study, before implementation of the PrEP program, has showed that non-western born MSM were more likely to have experienced difficulties accessing general healthcare and were less likely to have heard of post-exposure prophylaxis than non-migrant MSM (5). In our study, non-western born MSM did not have lower PrEP use, although it should be noted that we evaluated PrEP use among MSM who already accessed STI clinic care. Therefore, it is possible that non-western born migrant MSM were underrepresented in the clinic populations. Further, in line with previous studies (6, 7), we revealed sociodemographic disparities in PrEP use among non-western born MSM. PrEP use was lower among younger people, people with a low educational level, and people living in less urban areas. Previous studies among MSM have showed that higher income and a better perceived financial situation are associated with higher PrEP uptake (8, 9), indicating the importance of reimbursement of PrEP medication. MSM who live in less urban areas may face barriers to accessing HIV prevention, as shown for HIV testing (10). Having fewer peers in their social networks (role models) might play a role as peer network size has been associated with PrEP use in a previous study (6). International reports indicate that these findings regarding sociodemographic disparities in sexual healthcare use probably apply to western-born MSM as well, as is well known for many health issues and care access (11). Specifically, non-western born MSM who are younger, live in less urban areas or who have a low educational level might be vulnerable to accumulation of factors that are associated with less access to health-information and care options. These factors may reflect in language and financial barriers, administrative and housing insecurity, gender inequalities and discrimination, unawareness of health system in country of residence, and expected stigma and discomfort to talk about sexual health and HIV prevention (3, 12). Increasing availability (reducing current waiting lists) and fully reimbursing PrEP care are needed to improve access to PrEP for non-western born MSM with an increased risk for HIV, and hereby preventing avoidable disease burden and transmission of HIV. Additional strategies to increase PrEP uptake among non-western born MSM could be improving awareness of HIV prevention options, stimulating social approval and emotional support to overcome perceived fear and stigma. Participatory research could help to understand how increasing access and uptake could be organized in the community.

### 4.1. Strengths and limitations

This large study provides insight in PrEP use among non-western migrant MSM with a higher HIV risk and hereby underpins the importance of efforts to increase accessibility of PrEP to less reached groups. A limitation of this study was that the study population included only MSM visiting the STI clinics, which limits the generalizability to MSM who do not attend sexual health care. As PrEP care is mostly organized by STI clinics and less by general practitioners in the Netherlands, this study gives a good estimation of the absolute number of PrEP users, but likely overestimates the proportion of PrEP use among all non-western migrant MSM. Non-western born MSM are demonstrated to have less access to health care (in general). Other sampling methods, and inclusion of general practitioner data, are needed to estimate PrEP uptake among a wider group of non-western born MSM in the Netherlands. Another limitation of this study is that the PrEP care provider is unknown from the used registry data. Furthermore, we used a proxy for PrEP eligibility (based on number of sex partners and condom use during anogenital sex), as the PrEP eligibility criteria were unavailable from the standard registry data used in this study. Although this proxy ensured that we assessed PrEP use among MSM at risk for HIV, it is unclear whether this proxy would underestimate or overestimate people eligible for PrEP (per routine triage and practice). Finally, on the sociodemographic factor educational level, more missing values (categorized in unknown) were registered than among MSM born in the Netherlands. One explanation could be that the question about education in the current demographic/sexual history questionnaire used at Dutch STI clinics is not applicable to people who have followed education in non-western countries.

### 4.2. Conclusion

Non-western born MSM have a higher proportion of new HIV diagnoses, and also more often have used HIV-PrEP in the past 3 months than western-born MSM. Still, PrEP use in non-western born MSM who already attend sexual health care is not optimal. Efforts to increase accessibility to PrEP are needed, with specific attention to non-western born MSM who are younger, live in less urban areas and are lower educated.

## Data availability statement

The data analyzed in this study is subject to the following licenses/restrictions: the data used were explicitly made available for this study by the National Institute for Public Health and Environment. Any data sharing requests should be directed to the National Institute for Public Health and Environment. Requests to access these datasets should be directed to soap@rivm.nl.

## Ethics statement

Ethical review and approval was not required for the study on human participants in accordance with the local legislation and institutional requirements. Written informed consent for participation was not required for this study in accordance with the national legislation and the institutional requirements.

## Author contributions

YE, CG, CH, and ND-M contributed to the conception and design of the study. YE drafted the report and performed the statistical analyses. CG and ND-M contributed to statistical analysis. All authors contributed to the article and approved the submitted version.

## Conflict of interest

The authors declare that the research was conducted in the absence of any commercial or financial relationships that could be construed as a potential conflict of interest.

## Publisher’s note

All claims expressed in this article are solely those of the authors and do not necessarily represent those of their affiliated organizations, or those of the publisher, the editors and the reviewers. Any product that may be evaluated in this article, or claim that may be made by its manufacturer, is not guaranteed or endorsed by the publisher.

## Footnotes

^1^ www.cbs.nl
