# Supplementary material for: Newly diagnosed HIV and use of HIV-PrEP among non-western born MSM attending STI clinics in the Netherlands: a large retrospective cohort study
Source: Front Public Health. 2023 Jun 19;11:1196958. doi: 10.3389/fpubh.2023.1196958 (PMC10315834; doi:10.3389/fpubh.2023.1196958)
Supplement: Supplementary file 1 [file Table_1.docx]

Supplementary Material

Newly diagnosed HIV and use of HIV-PrEP among non-western born MSM attending STI clinics in the Netherlands: a large retrospective cohort study

Dr. Ymke J Evers^1,2^, Cornelia JD Goense^1,2^, Prof. dr. Christian JPA Hoebe^1,2,3^, Dr. Nicole HTM Dukers-Muijrers^1,4^

*** Correspondence:** [ymke.evers@ggdzl.nl](mailto:ymke.evers@ggdzl.nl)

# Table: sensitivity analysis

New HIV diagnoses among non-western born MSM, compared between demographic groups in subset of data (most recent consultations of non-western born MSM in the period between August 1, 2019 and December 31, 2021)

|  | New HIV diagnosis  N=58/4128  % (n/N) | OR |
| --- | --- | --- |
| Age group  <25  25-35  >35 | 2.1 (12/580)  1.3 (27/2128)  1.3 (19/1420) | 1.6 (0.8-3.2)  0.9 (0.5-1.7)  1 (ref) |
| Urbanicity  Low  High | 2.6 (10/381)  1.3 (48/3747) | 2.1 (1.0-4.1)*  1 (ref) |
| Educational level  Low  High  Unknown | 1.7 (18/1065)  0.7 (16/2432)  3.8 (24/631) | 2.6 (1.3-5.1)**  1 (ref)  5.9 (3.1-11.2)*** |
| Continent of birth  Latin America  Suriname/Dutch Antilles  Eastern Europe  Sub-Saharan Africa  North Africa  Asia | 3.3 (31/949)  1.5 (9/588)  1.1 (9/809)  1.2 (3/249)  0.0 (0/327)  0.6 (6/1206) | NA |
